# Spread of Carbapenem-Resistant Klebsiella pneumoniae in an Intensive Care Unit: A Whole-Genome Sequence-Based Prospective Observational Study

**DOI:** 10.1128/spectrum.00058-21

**Published:** 2021-07-14

**Authors:** Li Wei, Linfei Wu, Hongxia Wen, Yu Feng, Shichao Zhu, Ying Liu, Li Tang, Emma Doughty, Willem van Schaik, Alan McNally, Zhiyong Zong

**Affiliations:** a Department of Infection Control, West China Hospital, Sichuan University, Chengdu, China; b Respiratory/Infection Intensive Care Unit, West China Hospital, Sichuan University, Chengdu, China; c Center for Pathogen Research, West China Hospital, Sichuan University, Chengdu, China; d Center of Infectious Diseases, West China Hospital, Sichuan University, Chengdu, China; e Division of Infectious Diseases, State Key Laboratory of Biotherapy, Chengdu, China; f Institute of Microbiology and Infection, College of Medical and Dental Sciences, University of Birmingham, Birmingham, United Kingdom; Memorial Sloan Kettering Cancer Center

**Keywords:** carbapenemases, carbapenem resistance, *Klebsiella pneumoniae*, carbapenem-resistant *Enterobacteriaceae*, transmission, ICU antimicrobial resistance, carbapenems

## Abstract

The aim of this study was to determine the contribution of the contamination of the health care environment in the acquisition of carbapenem-resistant Klebsiella pneumoniae (CRKP) in a CRKP-prevalent setting. We performed a 3-month prospective study in a 20-bed medical intensive care unit (ICU) by collecting rectal/oral swabs from patients within 3 days of ICU admission and weekly thereafter. We also comprehensively sampled the beds and rooms of patients and instruments for patient care every week. CRKP were detected, genome sequenced, and assigned to clones based on core genome analyses. The survival of four CRKP clones was determined under ICU conditions. Seventeen patients were in the ICU at the start of the study, and 99 were admitted afterwards. Six were positive patients, with four detected on initial screening and two during weekly monitoring. CRKP was detected from 76 of 3,699 (2.1%) environment samples, including from the immediate surroundings of 21 patients (five had CRKP from clinical samples and 16 did not). CRKP was not detected outside patient care areas. Among 49 CRKP sequenced isolates (nine from swabs, five from clinical samples, and 35 from environment) from 21 patients, 45 were ST11 and had *bla*_KPC-2_. These could be assigned to four clones, with either KL47 (*n* = 22) or KL64 (*n* = 23) capsular type. The two dominant clones survived >30 days under ICU conditions. In conclusion, environmental contamination of CRKP was extensive but usually transient. It had little impact on CRKP acquisition by ICU patients, highlighting the ability to control CRKP transmission through infection prevention efforts even in high-prevalence settings.

**IMPORTANCE** Klebsiella pneumoniae can be an opportunistic pathogen with the oral cavity and gut the main origin. However, carbapenem-resistant Klebsiella pneumoniae (CRKP) can be found in patient surroundings and is a serious threat for human infections. Although the hospital environment, particularly sinks, has long been considered a potential reservoir of CRKP, the exact role of environmental contamination contributing to the acquisition and transmission of CRKP among patients remains largely unknown. To understand the link between environmental contamination in health care settings and colonization and infection of patients by CRKP, we performed a 3-month prospective study in a 20-bed medical ICU. Isolates were collected by active patient screening and were subsequently genome sequenced to describe the diversity of CRKP and the linkage of patients and environmental reservoirs. We found that the environmental contamination of CRKP was extensive, and CRKP clones were freely circulating in the ICU. Environmental contamination was not due to sharing the bed unit or sharing contaminated instruments but more likely resulted from the movement of health care workers. Very few patients acquired CRKP in the ICU, which is likely due to the fact that environmental contamination was usually transient when a routine cleaning protocol was complied. Although CRKP contamination in patient surroundings may be extensive, as long as routine environment cleaning protocols are appropriate and well implemented, the health care environment is unlikely to be a major source of CRKP colonization and infection in ICU patients. Reducing the high workload for ICU nurses may help minimize CRKP environmental contamination.

## INTRODUCTION

Carbapenem-resistant Klebsiella pneumoniae (CRKP) has been labeled a critical priority pathogen that poses a serious threat to human health by the World Health Organization (1). CRKP is largely associated with hospital-acquired infections and is particularly common in intensive care units (ICUs) (2, 3). There are very limited therapeutic options against CRKP, and it is therefore of considerable importance to prevent its transmission in health care settings such as ICUs (4). K. pneumoniae mainly resides in the human oral cavity and gut and is generally considered an opportunistic pathogen. Patients with CRKP can contaminate their surroundings, which may impose potential risks for other patients (5, 6). The hospital environment has long been considered a potential reservoir of CRKP (7), with sinks being linked to multiple outbreaks (8, 9). The exact role of environmental contamination contributing to the acquisition and transmission of CRKP among patients remains largely unknown (4).

**FIG 1:**
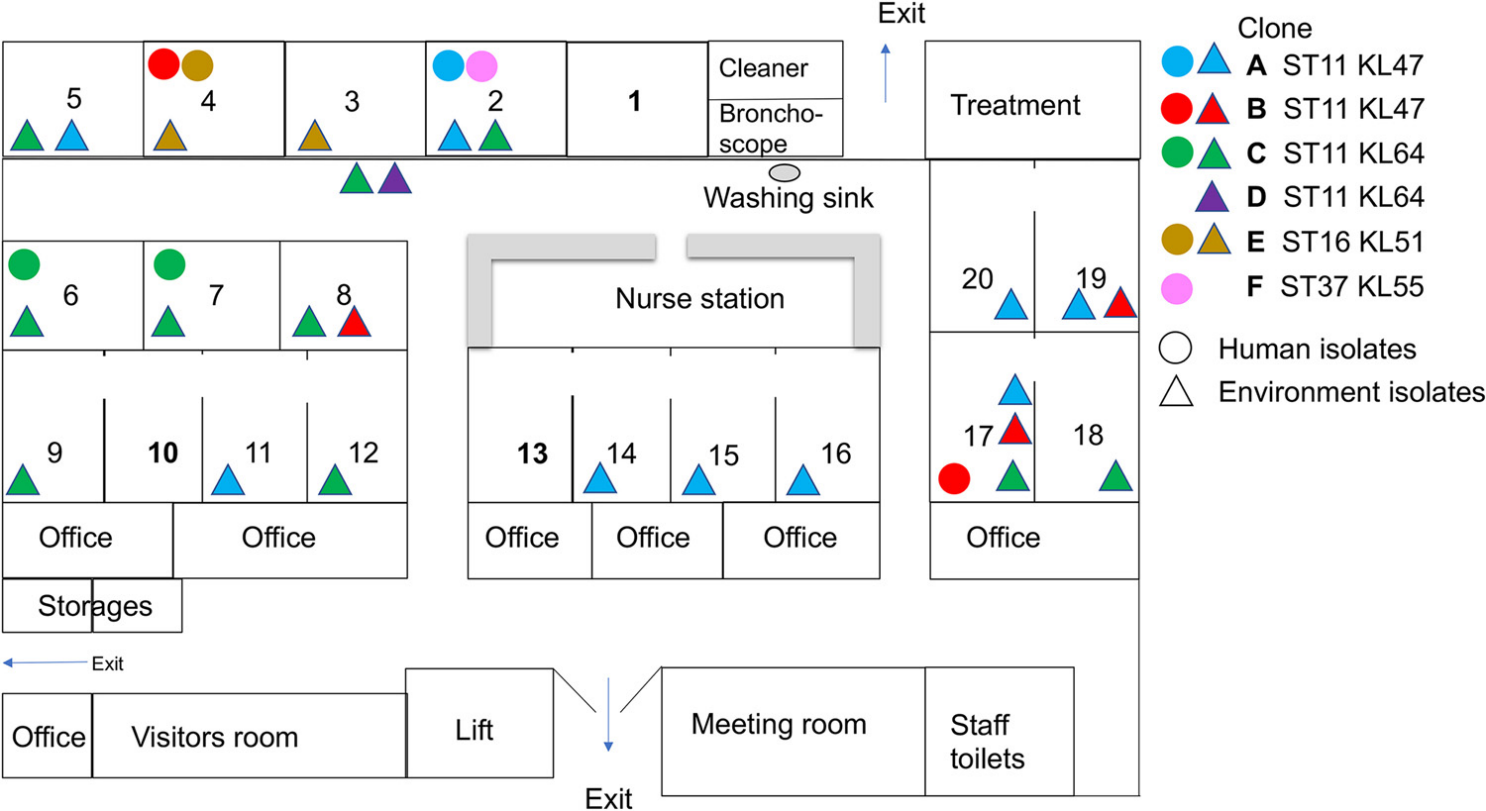
Plan of the ICU and the overall distribution of CRKP clones. There are 20 beds with 8 single-bed rooms (bed no. 1 to 8), two two-bed rooms (bed no. 17 and 18 and 19 and 20), and two four-bed rooms (bed no. 9 to 12 and 13 to 16). In the two- and four-bed rooms, each bed was located in a cubic with one or two glass walls to separate each other. The distribution of CRKP clones was superimposed by different time points during the study period. Clones A and B are shown in different colors, while human and environment isolates are depicted by circles and triangles, respectively.

To understand the link between environmental contamination in health care settings and colonization and infection of patients by CRKP, we performed a prospective observational study in a 20-bed medical ICU at West China Hospital, which has 5,000 beds and is the major referral medical center in western China (Fig. 1). Isolates were collected by active patient screening and were subsequently genome sequenced to describe the diversity of CRKP and the linkage of patients and environmental reservoirs.

## RESULTS

### CRKP carriage was limited upon admission and few patients developed CRKP infection during the ICU stay.

**FIG 2:**
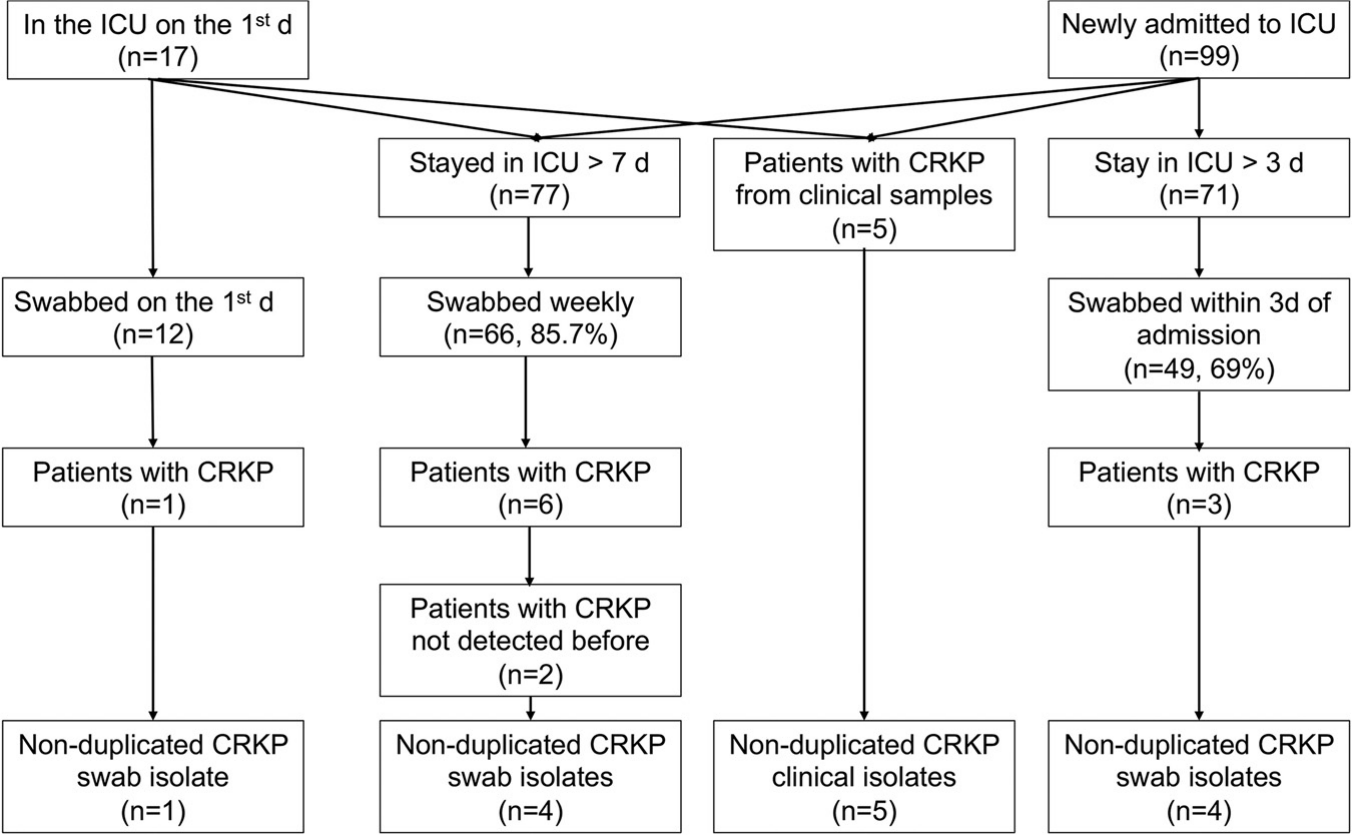
Flow chart of the study. For the purpose of selecting isolates for whole-genome sequencing, CRKP isolates from rectal and oral swabs of the same patient were regarded as nonduplicated in this study, while isolates from the same swab type of the same patient were regarded as duplicated. Patients without CRKP detected before are those who had no CRKP on admission (for patients newly admitted to the ICU) or on the first day of the ICU (for patients already stayed in the ICU prior to the study).

On the first day of the study, there were 17 patients in the ICU (Fig. 2). Rectal and oral swabs were collected from 12 of the 17 patients and CRKP was detected from only one patient (patient P7, from a rectal swab; Table 1) who had stayed in the ICU for 35 days before the study. During the study period, a total of 99 patients were newly admitted to the ICU, among whom 71 stayed in the ICU for more than 3 days (Fig. 2). Rectal and oral swabs were collected from 49 of the 71 patients (69.0%) within 3 days of admission. CRKP was detected from three patients, among whom one (P17-1, positive at the second ICU day) had CRKP from both oral and rectal swabs and the other two (P2-1 and P2-2, both were positive on the first ICU day) had CRKP from rectal swabs only. The CRKP carriage rate on admission was 6.1% (3/49).

**TABLE 1:** CRKP isolates in the present study^*a*^

Isolate no.	Sample type	Mo	Day	Bed no.	Patient no.^*b*^	ST	Clone	K type	Carbapenemase	ESBL and AmpC	MER MIC, mg/liter	GenBank accession no.
**090357**	**Rectal swab**	**6**	**11**	**7**	**P7**	**11**	**C**	**KL64**	**KPC-2**	**CTX-M-65**	**256**	**JACEYB000000000**
090352	Bedrail	6	12	7	P7	11	C	KL64	KPC-2	CTX-M-65	128	JACEXY000000000
090354	Ventilator panel	6	12	7	P7	11	C	KL64	KPC-2	CTX-M-65	128	JACEYA000000000
**090329**	**Oral swab**	**6**	**18**	**17**	**P17-1**	**11**	**B**	**KL47**	**KPC-2**	**CTX-M-65**	**64**	**JACEXV000000000**
**090360**	**Rectal swab**	**6**	**18**	**17**	**P17-1**	**11**	**B**	**KL47**	**KPC-2**	**CTX-M-65**	**256**	**JACEYE000000000**
**090466**	**Tracheal aspiration**	**6**	**18**	**17**	**P17-1**	**11**	**B**	**KL47**	**KPC-2**	**CTX-M-65**	**128**	**JACEZI000000000**
090359	Cardiovascular monitor panel	6	18	17	P17-1	11	B	KL47	KPC-2	CTX-M-65	256	JACEYD000000000
090358	Cardiovascular monitor panel	6	18	19	P19-1	11	B	KL47	KPC-2	CTX-M-65	128	JACEYC000000000
**090361**	**Rectal swab**	**6**	**19**	**2**	**P2-1**	**11**	**A**	**KL47**	**KPC-2**	**CTX-M-65**	**256**	**JACEYF000000000**
090362	Sink	6	26	8	P8	11	B	KL47	KPC-2	CTX-M-65	256	JACEYG000000000
**090465**	**Tracheal aspiration**	**7**	**2**	**7**	**P7**	**11**	**C**	**KL64**	**KPC-2**	**CTX-M-65**	**128**	**JACEZH000000000**
090363	Bed air pump	7	2	2	P2-1	11	A	KL47	KPC-2	CTX-M-65	128	JACEYH000000000
**090469**	**Sputum**	**7**	**8**	**2**	**P2-1**	**11**	**A**	**KL47**	**KPC-2**	**CTX-M-65**	**128**	**JACEZJ000000000**
**090472**	**Sputum**	**7**	**8**	**6**	**P6**	**11**	**C**	**KL64**	**KPC-2**	**CTX-M-65**	**128**	**JACEZK000000000**
**090346**	**Rectal swab**	**7**	**9**	**6**	**P6**	**11**	**C**	**KL64**	**KPC-2**	**CTX-M-65**	**128**	**JACEXW000000000**
**090348**	**Oral swab**	**7**	**9**	**6**	**P6**	**11**	**C**	**KL64**	**KPC-2**	**CTX-M-65**	**128**	**JACEXX000000000**
090373	Locker	7	9	15	P15	11	A	KL47	KPC-2	CTX-M-65	128	JACEYM000000000
090353	Bedrail	7	9	6	P6	11	C	KL64	KPC-2	CTX-M-65	128	JACEXZ000000000
090365	Ventilator panel	7	9	6	P6	11	C	KL64	KPC-2	CTX-M-65	128	JACEYI000000000
090366	Sink	7	9	6	P6	11	C	KL64	KPC-2	CTX-M-65	256	JACEYJ000000000
090371	Infusion pump	7	9	8	P8	11	C	KL64	KPC-2	CTX-M-65	4	JACEYK000000000
090372	Air-purifying disinfector panel	7	9	9	P9	11	C	KL64	KPC-2	CTX-M-65	128	JACEYL000000000
090374	Noninvasive ventilator panel	7	9	Corridor	—	11	D	KL64	KPC-2	CTX-M-15, CTX-M-65	256	JACEYN000000000
**090375**	**Oral swab**	**7**	**10**	**20**	**P2-1** ^*c*^	**11**	**A**	**KL47**	**KPC-2**	**CTX-M-65**	**256**	**JACEYO000000000**
**090376**	**Rectal swab**	**7**	**12**	**2**	**P2-2**	**37**	**F**	**KL55**	**-**	**CTX-M-27, DHA-1**	**128**	**JACEYP000000000**
090381	Bedrail	7	17	11	P11	11	A	KL47	KPC-2	CTX-M-65	128	JACEYS000000000
090382	Cardiovascular monitor panel	7	17	12	P12	11	A	KL47	KPC-2	CTX-M-65	256	JACEYT000000000
090539	Stethoscope	7	17	14	P14	11	A	KL47	KPC-2	CTX-M-65	128	JACEZQ000000000
090384	Nurse cart	7	17	16	P16	11	A	KL47	KPC-2	CTX-M-65	128	JACEYU000000000
090385	Nebulizer	7	17	17	P17-2	11	A	KL47	KPC-2	CTX-M-65	128	JACEYV000000000
090386	Hanging tower	7	17	17	P17-2	11	C	KL64	KPC-2	CTX-M-65	256	JACEYW000000000
090387	Nurse cart	7	17	18	P18-1	11	C	KL64	KPC-2	CTX-M-65	256	JACEYX000000000
090388	Bedrail	7	17	19	P19-2	11	A	KL47	KPC-2	CTX-M-65	256	JACEYY000000000
090389	Nebulizer	7	17	19	P19-2	11	A	KL47	KPC-2	CTX-M-65	128	JACEYZ000000000
090377	Air-purifying disinfector panel	7	17	2	P2-2	11	C	KL64	KPC-2	CTX-M-65	128	JACEYQ000000000
090391	Infusion pump	7	17	20	P2-1^*c*^	11	A	KL47	KPC-2	CTX-M-65	128	JACEZA000000000
090378	Nurse cart	7	17	5	P5	11	A	KL47	KPC-2	CTX-M-65	128	JACEYR000000000
090394	Bed air pump	7	23	8	—	11	C	KL64	KPC-2	CTX-M-65	128	JACEZB000000000
090547	Bed air pump	7	30	12	—	11	C	KL64	KPC-2	CTX-M-65	128	JACEZR000000000
090400	Bedrail	7	30	18	P18-2	11	C	KL64	KPC-2	CTX-M-65	256	JACEZC000000000
090401	Cardiovascular monitor panel	7	30	18	P18-2	11	C	KL64	KPC-2	CTX-M-65	256	JACEZD000000000
090458	Hanging tower	8	6	5	—	11	C	KL64	KPC-2	CTX-M-65	128	JACEZE000000000
090460	Nurse cart	8	6	Corridor	—	11	C	KL64	KPC-2	CTX-M-65	256	JACEZF000000000
090461	Noninvasive ventilator panel	8	6	Corridor	—	11	C	KL64	KPC-2	CTX-M-65	128	JACEZG000000000
**090483**	**Rectal swab**	**8**	**20**	**4**	**P4**	**11**	**B**	**KL47**	**KPC-2**	**CTX-M-65**	**256**	**JACEZP000000000**
090480	Bedrail	8	20	17	P17-3	11	A	KL47	KPC-2	CTX-M-65	256	JACEZN000000000
090475	Light switch	8	20	3	P3	16	E	KL51	NDM-5	CTX-M-15	128	JACEZL000000000
090479	Infusion pump	8	20	4	P4	16	E	KL51	NDM-5	CTX-M-15	256	JACEZM000000000
**090482**	**Sputum**	**8**	**25**	**4**	**P4**	**16**	**E**	**KL51**	**NDM-5**	**CTX-M-15**	**256**	**JACEZO000000000**

a Samples from patients including rectal and oral swabs and clinical samples are highlighted in boldface.

b The patient number was designated according to the bed number. For patients staying in the same bed but at different time points, the patient number was designated based on chronological order. For instance, P2-1 and P2-2 refer to two patients, but P2-1 stayed in bed no. 2 earlier than P2-2. —, there was no patient in the bed on the sampling day or instruments in the corridor could not be assigned to patients.

c Patient P2-1 had been transferred to bed no. 20.

A total of 77 patients were swabbed for weekly follow-up screening. We were able to collect rectal and oral swabs from 66 of the 77 patients (85.7%) during weekly surveillance. CRKP was detected from six patients (Fig. 2 and Table 1). Three of these patients (P2-1, P2-2, and P17-1) had CRKP-positive swabs on admission, and one (P7) stayed in the ICU before the study and had a CRKP-positive swab on the first day of the study. Two patients appeared to acquire CRKP in the ICU. One patient (P6) had the first swab collected at the fourth ICU day but had CRKP from a clinical sample collected at the third ICU day (see below). The remaining patient (P4) had no CRKP from swabs on admission but was found to be orally colonized at the eighth ICU day.

During the study period, five patients had CRKP isolated from clinical samples, either sputum (*n* = 3; P2-1, P4, and P6) or tracheal aspirates (*n* = 2; P7 and P17-1). All five patients had CRKP-positive rectal or oral swabs within 1 day before or after the collection of the CRKP-positive clinical sample. All patient isolates were submitted for genome sequencing.

### Extensive contamination of CRKP in patient surroundings.

A total of 3,699 environmental samples were collected, including 2,964 from the surrounding of 82 patients. CRKP was detected from 76 samples (positive rate, 2.1%), including 67 from the immediate surroundings of 21 patients, six from two sinks in patient rooms, and three from the surface of instruments (two noninvasive ventilators and one nurse cart) in the corridor outside patient rooms (Table 2 and Fig. 1). These CRKP isolates were detected at one or more sites from the surroundings of 21/82 (25.6%) patients occupying 17 of the 20 (85.0%) beds. Except for the three instruments in the corridor, CRKP was not detected in any samples collected from areas outside patient rooms.

**TABLE 2:** Environment sampling results

Area	Surface type^*a*^	Sampling site	Sampling no.	No. positive	% Positive
Patient care area					
Bed surroundings	Noninstrument	Bedrails	257	12	4.7
Bed surroundings	Noninstrument	Hanging towers	257	5	2.0
Bed surroundings	Noninstrument	Light switches	257	2	0.8
Bed surroundings	Noninstrument	Bed air pumps	255	4	1.6
Bed surroundings	Noninstrument	Lockers	255	5	2.0
Bed surroundings	Noninstrument	Stethoscopes	253	6	2.4
Bed surroundings	Instrument	Cardiovascular monitors	257	4	1.6
Bed surroundings	Instrument	Air-purifying disinfectors	249	3	1.2
Bed surroundings	Instrument	Infusion pumps	248	9	3.6
Bed surroundings	Instrument	Ventilators	239	6	2.5
Bed surroundings	Instrument	Nurse carts	234	6	2.6
Bed surroundings	Instrument	Nebulizers	203	5	2.5
Patient rooms		Computer mouse/keyboards	150	0	0.0
Patient rooms		Drinking water dispenser buttons	130	0	0.0
Patient rooms	Sink	Sink top surface	82	1	1.2
Patient rooms	Sink	Faucets	82	1	1.2
Patient rooms	Sink	Internal surfaces	82	3	3.7
Patient rooms	Sink	Drains	82	1	1.2
Corridor	Instrument	Vibration sputum ejection machines	10	0	0.0
Corridor	Instrument	Emergency rescue carts	6	0	0.0
Corridor	Instrument	Noninvasive ventilators	6	2	33.3
Corridor	Instrument	Steppers	4	0	0.0
Corridor	Instrument	Nurse carts	3	1	33.3
Corridor	Instrument	Walk helpers	3	0	0.0
Outside patient area					
Nurse station	Sink	Sink top surface	3	0	0.0
Nurse station	Sink	Faucets	3	0	0.0
Nurse station	Sink	Internal surfaces	3	0	0.0
Nurse station	Sink	Drains	3	0	0.0
Nurse station		Computer mouse/keyboards	9	0	0.0
Nurse station		Telephones	6	0	0.0
Nurse station		Barcode scanners/printers	5	0	0.0
Nurse station		Medical record holder	3	0	0.0
Nurse station		Wireless interphones	3	0	0.0
Treatment room		Apparatus storage cabinets	25	0	0.0
Treatment room		Liquid storage cabinets	5	0	0.0
Treatment room		Refrigerator doorknobs	4	0	0.0
Treatment room		Light switches	3	0	0.0
Bronchoscope room		Bronchoscopes	5	0	0.0
Bronchoscope room		Drying tabletop	3	0	0.0
Bronchoscope room		Bronchoscope cabinet doorknob	3	0	0.0
Bronchoscope room		Bronchoscope cabinet internal wall	3	0	0.0
Bronchoscope room		Washing machine buttons	3	0	0.0
Bronchoscope room		Internal of washing tanks	3	0	0.0
Cleaner room		Cleaner vehicle body	3	0	0.0
Cleaner room		Cleaner vehicle cap	3	0	0.0
Cleaner room		Cleaner vehicle hand shank	3	0	0.0
Cleaner room		Mop handles	3	0	0.0
Meeting room		Computer mouse/keyboards	7	0	0.0
Meeting room		Drinking water dispenser buttons	3	0	0.0
Staff toilets		Doorknobs	6	0	0.0
**Total**			**76**	**3,699**	**2.1**

a Instrument and noninstrument surfaces are assigned to surface types in the patient care area.

Among the 21 patients with CRKP detected in their surroundings, six had CRKP from their rectal/oral swabs and/or clinical samples. The interval between the first positive rectal/oral swab and the first positive environmental sample was 0 (the same day; patients P4, P6, P7, and P17-1), 5 days (patient P2-2), or 13 days (patient P2-1) (Table 1). For five of the six patients, CRKP was always detected from their environment in each weekly sampling during their entire ICU stay, suggesting continuous contamination. In particular, 33 out of the 76 CRKP-positive environment samples were recovered from multiple sites in the surroundings of a single patient (patient P6) across a period of 7 weeks. Among the different sampling sites around patient P6, the panel of the ventilator, sink, bedrail, and hanging tower remained CRKP positive for the whole period of 7 weeks during the ICU stay. In contrast, for the 15 patients without CRKP colonization or infection, CRKP was only detected once in the weekly environment sampling. This suggests that the environmental contamination for patients without CRKP colonization or infection is usually transient and restricted.

### Most CRKP isolates belonged to ST11, either the KL47 or KL64 capsular type.

A total of 49 CRKP isolates from 21 patients, including 9 from screening swabs (five from rectal swabs and four from oral), five from clinical samples, and 35 from the environment, were sequenced. The vast majority of the 49 CRKP isolates belonged to ST11 (*n* = 45, 91.8%), either to the KL47 (*n* = 22) or KL64 (*n* = 23) capsular type, while the remaining four belonged to ST16 KL51 (*n* = 3) and ST37 KL55 (*n* = 1). All of the ST11 isolates had the carbapenemase-encoding gene *bla*_KPC-2_ and an extended-spectrum β-lactamase (ESBL)-encoding gene *bla*_CTX-M-65_. The three ST16 CRKP isolates had the carbapenemase-encoding gene *bla*_NDM-5_ and an ESBL-encoding gene, *bla*_CTX-M-15_, while the ST37 isolate exhibited low-level resistance to meropenem (MIC, 4 mg/liter). No known carbapenemase-encoding genes were identified in this ST37 isolate, but its *ompK36* was interrupted by an insertion sequence of the IS*5* family. This isolate also had an ESBL-encoding gene, *bla*_CTX-M-27_, and an AmpC-type cephalosporinase-encoding gene, *bla*_DHA-1_. The combination of OmpK36 alteration and production of ESBL or AmpC has previously been reported to lead to low-level carbapenem resistance (10–12).

### Interpatient transmission and environmental contamination by ST11 KL47 KPC-2-producing CRKP clones.

**FIG 3:**
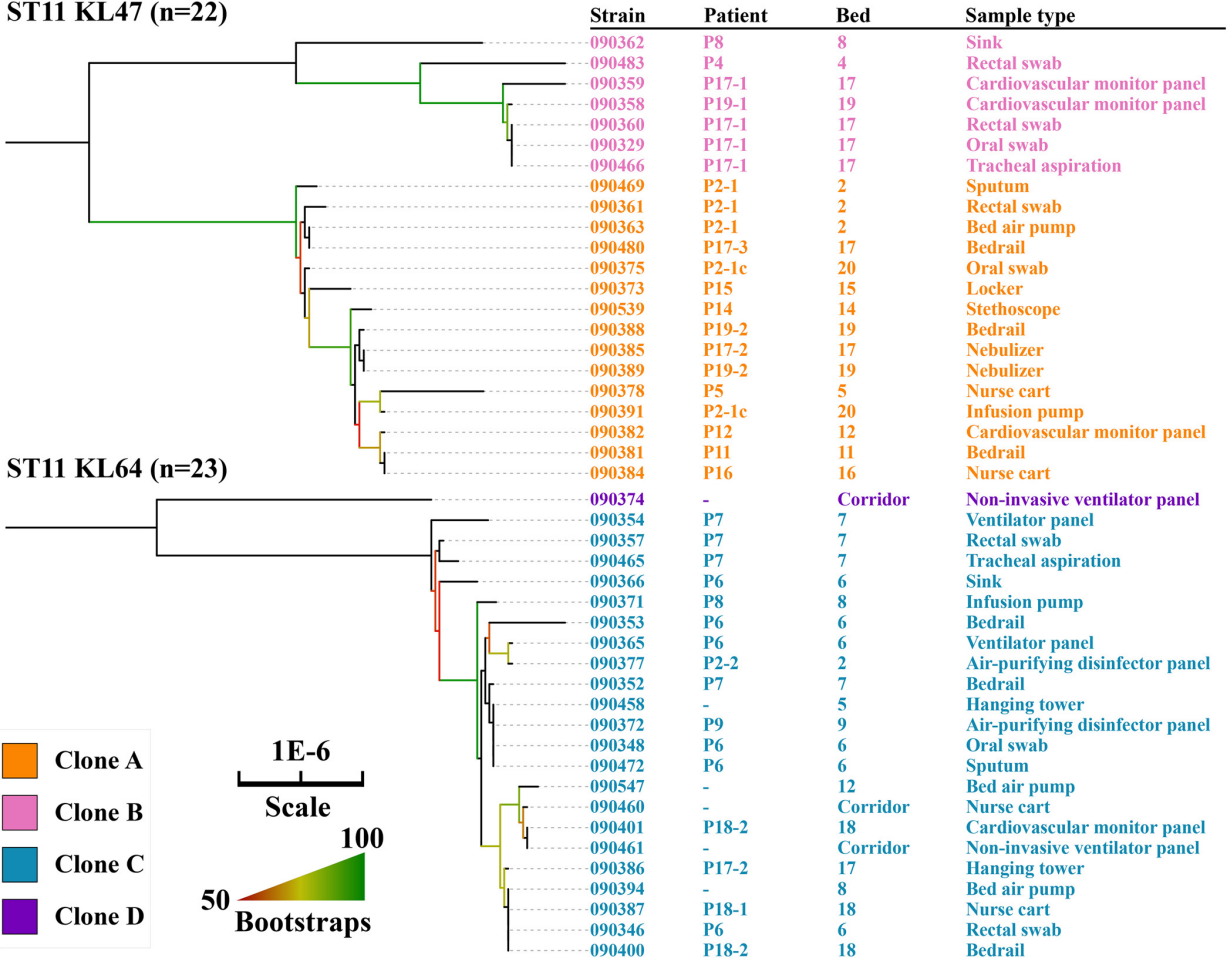
Phylogenomic trees of ST11 CRKP isolates. Isolate name, patient number, bed number, and sample type (see Table 1 for details) are shown. All ST11 CRKP isolates had *bla*_KPC-2_.

The 22 ST11 KL47 KPC-2-producing CRKP isolates could be assigned to two clones based on single-nucleotide polymorphism (SNP) analysis (Fig. 3). Clone A consisted of 15 isolates associated with 10 patients, with 0 to 13 SNPs between them. Five isolates were recovered from a single patient (P2-1) from the rectal and oral swabs, a clinical sample (sputum), an instrument, and a non-instrument surface. The first isolate from clone A originated from a rectal swab of patient P2-1 that was collected on admission day, providing evidence that the clone was introduced into the ICU by this patient. A sputum sample from this patient with this clone was collected 16 days after admission, suggesting that the patient developed a health care-associated infection after gut colonization. The first appearance of this clone in the environment of the ICU was 13 days after admission of this patient, and subsequently clone A was also detected in the surroundings of 9 other patients scattered across the ICU in different beds (P5, P11, P12, P14, P15, P17-1, P17-2, and P19) across a period of 9 weeks (Fig. 1), suggesting extensive and prolonged contamination of the ICU by this single clone.

Clone B comprised seven isolates associated with four patients with 0 to 21 SNPs between them but which differed from the isolates in clone A by 27 to 44 SNPs (see Data Set S1 in the supplemental material). The first four isolates were from the same patient (P17-1) and were collected on the second day of ICU admission. The CRKP-positive instrument surface sample of this patient was collected on the same day, suggesting rapid contamination of CRKP from the patient to the environment. Clone B then was also found on an instrument surface of another patient (P19) and in a sink in the room of P8 (Fig. 1). In addition, an isolate of clone B was identified from an oral swab of patient P4 9 weeks after the first isolation of the clone, suggesting prolonged transmission of the clone in the ICU.

### The hospital acquisition and environment contamination of an ST11 KL64 KPC-2-producing CRKP clone.

Twenty-two of 23 ST11 KL64 KPC-2-producing CRKP isolates were assigned to clone C, as their core genomes differed by 0 to 9 SNPs (Data Set S2), while the remaining ST11 KL64 KPC-2-producing CRKP isolate had 30 to 36 SNPs compared to clone C (Data Set S2). Isolates of clone C were recovered from samples or the environment of seven patients, the environment of three other unoccupied beds, and three instruments in the corridor across a period of 7 weeks. This suggests extensive environment contamination of this clone in the ICU. The first isolate of clone C was recovered from a rectal swab of a patient (P7) on the first day of the study, suggesting that the clone had already been in the ICU before the start of the study. Isolates of clone C were also found in sputum, rectal, and oral swabs from another patient (P6) 26 days after study initiation. This shows a hospital acquisition from either patient P7 or the contaminated environment (Fig. 1).

### Environmental contamination of a ST16 KL51 NDM-5-producing CRKP clone.

There were no SNPs among the three ST16 isolates. The three isolates were recovered from a clinical sample (sputum) and an instrument surface (an infusion pump) of one patient (P4) and from a noninstrument surface (a light switch) of another patient (P3). The two patients were in two single rooms next to each other (Fig. 1), suggesting environment contamination and transmission of the CRKP clone. The isolate from the instrument surface was recovered 5 days previous to that from sputum of the same patient. Therefore, it is more likely that the patient had acquired the clone from the environment rather than vice versa. The three isolates were recovered in August, 2 months after the study start, suggesting that the ST16 NDM-5-producing strain was introduced into the ICU through an unknown source.

### Isolates of clone C and clone B exhibited prolonged environmental survival under ICU conditions.

In an *in vitro* survival assay, isolates of the four ST11 clones (clone A to D) could survive in the real ICU situation (temperature of 24 to 28°C, relative humidity of 56% to 60%) for 32 to >43 days, except for one clone A isolate (for 22 days), while non-ST11 CRKP isolates had a ≤30-day survival (Table 3). All three clone C isolates survived under ICU conditions for > 43 days, which was longer than those of isolates of clone A (22 to 40 days) or clone D (37 days), and also survived longer than at least one clone B isolate tested (22 days) (Table 3). The relatively prolonged survival of clone C isolates may contribute to its wide distribution in the ICU.

**TABLE 3:** *In vitro* survival of CRKP isolates in the ICU setting

ST, KL type	Clone	Isolate	Survival, days
11, KL47	A	090361	32
	A	090381	22
	A	090480	40
11, KL47	B	090359	>43
	B	090362	34
	B	090483	>43
11, KL64	C	090357	>43
	C	090353	>43
	C	090461	>43
11, KL64	D	090374	37
16, KL51	E	090475	20
	E	090479	26
	E	090482	24
37, KL55	F	090376	30
E. coli		ATCC 25922	4
*K. quasipneumoniae*		ATCC 700603	20

## DISCUSSION

In the present study, we found extensive environmental contamination of CRKP in the ICU. Among the 21 patients whose surroundings contained CRKP, 14 (2/3) stayed in a bed unit from which no CRKP was recovered from other patients who had stayed in the same bed unit at different time points. The remaining seven patients stayed in three different bed units, from which CRKP was recovered from the surroundings of two or three patients who stayed in the same bed at different time points. Thus, sharing of a bed unit was not an important driver for extensive contamination of CRKP in the ICU. The same clone was repeatedly seen in the surroundings of different bed units, suggesting that CRKP clones were freely circulating in the ICU. The exact medium of the spread of CRKP clones remains unclear but is likely due to the movement of health care workers in the ICU. There are 17 doctors, 54 nurses, 13 technicians, three workers, and two cleaners in the ICU. The high workload, in particular for ICU nurses, may lead to reduced compliance with infection control measures such as hand hygiene. Previous studies have demonstrated that hands, gloves, and gowns of ICU staff may be contaminated by CRKP (5, 13) and are likely to mediate its spread. This highlights the importance of hand hygiene and the need to promptly change gloves and gowns after contact patients with CRKP as part of the infection control bundle against carbapenem-resistant organisms (4). The current World Health Organization (WHO) guideline does not recommend screening of health care workers for carriage of carbapenem-resistant organisms (4), and more studies may be needed to demonstrate the true value of this exercise in both outbreak and nonoutbreak settings. Sharing contaminated instruments may be another factor to contribute to the extensive environment contamination. However, most instruments, such as ventilators, infusion pumps, and monitors, were dedicated to the corresponding bed units, and our data clearly demonstrated that these instruments were not the source of dissemination.

ST11 KPC-2-producing CRKP can survive at room temperature and humidity encountered in the ICU for more than 43 days, as determined using the *in vitro* survival assay. However, CRKP was almost always detected only once from the surroundings of patients without CRKP colonization or infection and was not detected in the next round of sampling, which was 7 days later. This indicates that the CRKP isolates were removed by routine cleaning in the ICU, which could have significantly reduced the risks of patients acquiring CRKP. The routine environment cleaning protocol in this ICU included using wipes moistened with 70% ethanol for instrumental surfaces or with 500 mg/liter chlorine for other surfaces, such as bedrails, at a frequency of twice daily. In this study, only two patients (P4 and P6) acquired CRKP despite the extensive contamination of the environment (Fig. 1). In these two patients, their first CRKP-positive environmental samples were collected on the same day as, or 1 day later than, their screening swabs or clinical samples. Given that environmental contamination was so transient, this suggests that CRKP isolates from the patients contaminated the environment.

There are a few potential limitations to this study. The compliance of screening swabs on admission was around 70%. Some patients with CRKP carriage might have been missed in our screening. We also did not sample offices, storage rooms, and the visitor room of the ICU. However, these locations likely present lower risks in mediating transmission of CRKP to patients, as none of the samples taken from outside patient care areas were positive for CRKP in this study.

### Conclusions.

Despite these factors, this study provides important insights into CRKP and the health care environment in ICUs. CRKP contamination in patient surroundings may be extensive, but as long as routine environmental cleaning protocols are appropriate and well implemented, the health care environment is unlikely to be a major source of CRKP colonization and infection in ICU patients. Awareness of infection prevention practices will remain crucially important to minimize the spread of CRKP.

## MATERIALS AND METHODS

### Prospective study into CRKP colonization.

This was a prospective investigation that was carried out during a 3-month period between June 2019 and September 2019 in a 20-bed medical ICU at West China Hospital. This study was approved by the Ethical Committee of West China Hospital, with informed consent being waived. Clinical samples were sent by clinicians if an infection was suspected based on their clinical judgements. Bed units were numbered in the ward. Patient identifiers were assigned according to their bed unit and order of patient occupation if more than one patient stayed in the bed during the study period, e.g., patients P2-1 and P2-2 both stayed in bed unit 2, first and second within the study, respectively).

Rectal and oral swabs (Haibo, Qingdao, China) were collected from all patients within 3 days of admission to the ICU and weekly during the ICU stay thereafter. All swabs were screened for CRKP using Simmon’s citrate agar plates (Haibo) (14, 15) supplemented with 1% inositol with the addition of 2 mg/liter meropenem.

### Environmental sampling.

In patient rooms, for each bed unit comprising a patient bed and its surroundings, we performed weekly sampling for the entire surface of all bedrails (right, left, head, and foot), the light switch, the stethoscope, the locker handle and buttons of the bed air pump, the button panel of ventilators, cardiovascular monitors, infusion pumps, air-purifying disinfectors, nebulizers plus their hand shanks, the 1,200-cm^2^ (30 cm by 40 cm) surface of the hanging tower, and nurse carts plus the entire surface of handles of nurse cart drawers. For each patient room, we also sampled the entire surface of computer mouse/keyboards as well as buttons of the drinking water dispenser weekly. Each of the 12 ward rooms has a handwashing sink. All sinks, including faucets, all surfaces, including the entire internal surface of the bowl including overflows, and drains were sampled once every 2 weeks.

Instruments such as ventilators, nurse carts, emergency rescue carts, vibration sputum ejection machines, Zimmer frames, and steppers (step trainers) are often left in the corridor outside patient rooms for temporary storage or when awaiting repair. These were also sampled if present when weekly sampling was performed. Outside patient rooms, we performed monthly sampling of the nurse station, the bronchoscope room, the treatment room, the cleaner room, the meeting room, and staff toilets. For the nurse station, we sampled the entire surface of computer mouse/keyboards, telephones, wireless interphones, barcode scanners/printers, and the medical record holder. The handwashing sink close to the nurse station in the corridor was also sampled at four sites as described above. For the bronchoscope room, we sampled the entire surfaces of the drying tabletop, the handle, the internal wall of the bronchoscope storage cabinet, the button panel of the washing machine, and inside washing tanks. We also sampled the lumen of the three bronchoscopes in addition to their operating buttons once during the 3-month period. For the treatment room, we sampled the entire surfaces of the light switch, the doorknob of the refrigerator, and a 30-cm by 40-cm surface on the storage cabinets. For staff toilets, we sampled the entire surface of doorknobs. For the meeting room, we sampled the entire surface of computer mouse/keyboards and buttons of drinking water dispensers. For the cleaner room, we sampled the entire surface of mop handles and the hand shank of the cleaner vehicle and a 10-cm by 10-cm area of the vehicle’s lid and body, respectively.

Surfaces and sinks were sampled using sterile rayon swabs (Copan; Brescia, Italy) moistened with tryptic soy broth (TSB; Hopebio). The swabs from surfaces were streaked onto Simmon’s citrate agar plates (supplemented with 1% inositol) containing 2 μg/ml meropenem to screen CRKP. Swabs from sinks were immediately placed into 15-ml sterile tubes containing 6 ml TSB after sampling and were processed as described below. The lumen of bronchoscopes was washed using 50 ml phosphate neutralizing buffer containing 3 g/liter glycine, 10 g/liter peptone, 8.5 g/liter sodium chloride, and 1 g/liter Tween 80 (16), which was then passed through 0.22-μm filters (Millipore, Burlington, MA, USA). The filters were then incubated into 15-ml sterile tubes containing 6 ml TSB. The tubes were incubated at 37°C overnight and centrifuged. Supernatants were discarded and cell pellets were resuspended in 1 ml TSB. A 50-μl suspension aliquot was streaked onto Simmon’s citrate agar plates (supplemented with 1% inositol) containing 2 μg/ml meropenem.

### WGS and analysis.

For CRKP isolates from patients, the first isolate from each rectal swab, oral swab, and clinical sample was subjected to whole-genome sequencing (WGS). For CRKP isolated from environment sampling, up to three isolates were sequenced for each patient, including the first isolate from any instrument surface, the first isolate from any non-instrument (bedrails, bed air pumps, light switches, stethoscopes, locker handles, and hanging towers) surface, and the first isolate from any sink samples. Genomic DNA was prepared using the QIAamp DNA minikit (Qiagen, Hilden, Germany), and DNA sequencing libraries were prepared using the NEBNext Ultra II DNA library prep kit for Illumina (NEB, Ipswich, MA, USA). Reads were assembled into contigs using SPAdes v3.14.1 (17) with careful mode. Quality check was performed on the assembled genomes using CheckM v1.0.18 (18) to determine the existence of contamination. Sequence type (ST) was determined using the genome sequence to query the multilocus sequence typing database (http://bigsdb.pasteur.fr/klebsiella/klebsiella.html). Capsule (KL) typing and virulence factor prediction were performed using Kleborate v1.0.0 (19). Acquired antimicrobial resistance genes were identified using ResFinder (http://genomicepidemiology.org/). Gene sequences of outer membrane porins (OMPs) OmpK35- and OmpK36-encoding *ompK35* and *ompK36* of an ST37 CRKP isolate without known carbapenemase-encoding genes were compared with those of four carbapenem-susceptible ST37 isolates in our isolate collections to identity potential mechanism for carbapenem resistance by Clustal Omega (20).

### SNP calling.

SNPs among CRKP isolates of the same ST and KL type were determined by mapping individuals against the complete genome of strains 090329 and 090357 and the draft genome of strain 090482 for strains of ST11-KL47, ST11-KL64, and ST16-KL51, respectively, using Snippy v4.6.0 (https://github.com/tseemann/snippy). Pseudo-multialignments of chromosomes were generated using scripts provided by Snippy, followed by recombination prediction and filtering using Gubbins v2.4.1 (21) under the GTRGAMMA model with a maximum of 50 iterations. Pairwise SNP distances were calculated from recombination-free chromosome alignments using snp-dists v0.7.0 (https://github.com/tseemann/snp-dists). Although the exact genome mutation rate of CRKP is unknown, several previous studies have estimated that it ranges from 3.9 to 18.4 SNPs per genome per year (22, 23). Therefore, we used a cutoff of ≤21 high-quality core genome SNPs, as described previously (24), to define clones within the same ST and KL type.

### Antimicrobial susceptibility testing.

MICs of meropenem were determined using the broth microdilution method of the Clinical and Laboratory Standards Institute (CLSI) (25).

### Survival assay.

The survival of CRKP clones on polyvinyl chloride (PVC) surfaces at room temperature and humidity in the real ICU situation (temperature, 24 to 28°C; relative humidity, 56% to 60%) up to 43 days was performed as we described previously (26). Briefly, each strain was incubated to mid-exponential growth phase, centrifuged, washed, and resuspended in 1 ml phosphate-buffered saline (PBS) and finally adjusted to 0.5 MacFarland turbidity-equivalent standard (10^8^ CFU/ml). Aliquots (10 μl) of each strain were added in wells (three wells per strain) of a sterile PVC 96-well plate (Corning, New York). After drying in ambient air overnight in a clean biosafety cabinet, the plates were put into a paper box and were then placed in a corner of the corridor in the ICU away from patient care areas. ICU staff were notified of the place to avoid any potential touches. Bacterial cells were recovered by adding 200 μl PBS into each well, with shaking with 85 ± 5 rpm for 60 min by a miniature orbital shaker (BETS-M1; Qilinbeier, Haimen, Jiangsu, China), streaking a 100-μl suspension on LB agar, and incubating at 37°C overnight. For clones with three or more isolates (clones A, B, C, and E), three isolates of each clone were selected for the assay, while for clones with only one isolate (clones D and F), the sole isolate was included. Escherichia coli strain ATCC 29922 and Klebsiella quasipneumoniae strain ATCC 700603 (carbapenem-susceptible) were used as controls.

### Data availability.

The draft genomes of the isolates have been deposited in GenBank under accession no. JACEXV000000000–JACEZR000000000.
